# Generalization of DT Equations for Time Dependent Sources

**DOI:** 10.3390/s101210828

**Published:** 2010-12-02

**Authors:** Lorenzo Neri, Salvatore Tudisco, Francesco Musumeci, Agata Scordino, Giorgio Fallica, Massimo Mazzillo, Massimo Zimbone

**Affiliations:** 1 Laboratori Nazionali del Sud, Istituto Nazionale di Fisica Nucleare, via S. Sofia 62, 95123 Catania, Italy; E-Mails: tudisco@lns.infn.it (S.T.); musumeci@dmfci.unict.it (F.M.); ascordin@dmfci.unict.it (A.S.); 2 Università di Catania, via S. Sofia 64, 95123 Catania, Italy; E-Mail: massimo.zimbone@ct.infn.it (M.Z.); 3 ST-Microelectronics, Stradale Primosole 50, 95121 Catania, Italy; E-Mails: giorgio.fallica@st.com (G.F.); massimo.mazzillo@st.com (M.M.)

**Keywords:** Dead Time correction, time dependent sources, Single Photon Avalanche Diode

## Abstract

New equations for paralyzable, non paralyzable and hybrid DT models, valid for any time dependent sources are presented. We show how such new equations include the equations already used for constant rate sources, and how it’s is possible to correct DT losses in the case of time dependent sources. Montecarlo simulations were performed to compare the equations behavior with the three DT models. Excellent accordance between equations predictions and Montecarlo simulation was found. We also obtain good results in the experimental validation of the new hybrid DT equation. Passive quenched SPAD device was chosen as a device affected by hybrid DT losses and active quenched SPAD with 50 ns DT was used as DT losses free device.

## 1. Introduction

When using an electronic signal counter, the Dead Time (DT) must be considered, to be sure that the measurement is free from losses or that the losses have been correctly calculated. The DT is the time after the detection of an event, in which the acquisition system is not able to detect new events. On increasing the counting rate the DT effect on the total number of counts detected increases. The DT can be due or to the sensor device or to the electronics readout system. Theoretical studies of the DT behavior are often used to infer the real amount of count rate from the measured one [1]. The greater part of literature has studied the DT influence on stationary sources [2], that is when the intensity variations of the source occur in time interval order of magnitude longer than the DT of the counter device.

There are two elementary idealized models for DT, named paralyzable (P-DT) and non paralyzable (NP-DT). In the P-DT case all events occurring during the DT are not registered, but are able to prolong the period during which the detector is not able to reveal further signals. In the NP-DT case, any event arriving during the DT is neither registered nor has any influence on the device. In the real equipments parallel or series combinations of these two kinds of DT can be found. One of the most important configuration is the hybrid model [3], a series of NP-DT followed by a P-DT, used for Geiger-Muller (GM) devices.

The Avalanche Photon Diode (APD) can be used over the breakdown voltage like a GM device [4], with single photon sensitivity and excellent time performances: in such configuration the device is called Single Photon Avalanche Diode (SPAD). Recently we have demonstrated that the SPAD DT configuration can be considered like the GM one and that hybrid DT model can be used for passive quenched SPAD device [5].

Some studies have performed the analysis of short-lived time dependent source [6,7] but limited to the case of a well know shape dependency, for example in the case of decaying source with a single decay constant *λ* as *n*(*t*)*dt* = (*b* + *s*_0_*e*^−^*^λt^*)*dt*, where *n* is the time dependent event rate, *b* is the constant background rate, *s_0_* is the starting pulse rate. Nevertheless there are many different types of source and many case in which such sources are mixed together in a non predetermined way or have a non predetermined shape. The relevance of this problem motivates the developing of complex electronic systems for DT compensation as reported in [7], but such complex solutions cannot be used in any condition.

With our theoretical approach it is possible to evaluate the DT losses only by using a data analysis process, apart from the time dependence of the source, and without the necessity of developing complex electronic systems. In this work we present three new equations that describe the time dependent effect of paralyzable, non paralyzable, and hybrid DTs, and the accordance of the equations predictions with Montecarlo simulations and experimental data.

## 2. Materials

The goal of our study is to extend the dynamic range of our imaging device developed by using SPAD technology [8], and, in particular, passive quenched SPAD. The diodes used have been previously described [5], the DT configuration is hybrid model like, the total DT is 970 ns, 660 ns NP-DT and 310 ns P-DT. As a reference counter a commercial active quenching device with 50 ns of NP-DT was used. As a matter of fact at the maximum rate used in our experimental setup this device can be considered free from losses.

We realized a modulated light source using a commercial Light Emitting Diode (LED) powered by a pulse generator, model PB-4. The shape of the light source obtained is defined by an exponential grow, with a selectable characteristic time between 0.05 μs and 10 μs, and an exponential fall down with a selectable time between 0.5 μs and 1000 μs. Both counter devices were contemporaneously enlighten by the same light source. The duration of the acquisition was 35 μs and, to increase the statistics, about 10E6 run have been performed. The readout system has been designed to obtain an histogram of the measured count rate *vs*. time, with a time bins of 10 ns, even if the acquisition time precision is 0.1 ns.

## 3. New Dead Time Equations

### 3.1. Non Paralyzable Dead Time

As said, the signals that arrive during the NP-DT interval after a detected signal are lost. In the case of NP-DT the total inactive time of the sensor can be calculated by multiplying the measured count rate (*m*) and the DT extension (τ*_np_*). The losses count rate are the real incident rate (*n*) in the total inactive time. So in this case the measured count rate is related to the real incident rate by the equation:

(1) $$m=n\cdot (1-m\cdot \tau_{\text{np}})$$

that can be rewritten as:

(2) $$m=\frac{n}{1+n\cdot \tau_{\text{np}}}$$

Equation (2) is the commonly used equation to describe the NP-DT regime [1]. This equation is valid for constant rate sources, while in the case of time dependent sources the count rate *m* and *n* are functions of the time. Starting from this consideration we determine an equation valid for a time dependent count rates. We divide the total time in intervals of duration *Δ* and consider the average rate in each interval so obtaining two series of values *m_i_* and *n_i_*. With this time division the duration τ*_np_* turns into a number *T_np_* of intervals. For example, if it is τ*_np_* = 500 ns and the interval duration Δ = 10 ns, then *T_np_* = 50. Since the NP-DT losses are due to the measured counts in the previous DT interval (τ*_np_*), to evaluate the measured rate in the *i-th* interval we sum the contribute of all the previous *T_np_* measured rate values. Due to the finite duration of the *i-th* interval we must also consider the DT losses inside the same interval. The count rate at the end of the interval suffer of the losses due to the previous rate measured inside the same interval, whereas the rate at the first part of the interval are not influenced by the following part of the interval. So evaluating the average losses produced inside the interval, we obtain that the *i-th* interval must be considered like a source of DT losses, but half weighted. Applying these considerations to Equation (1) we obtain:

(3) $$m_{i}=n_{i}\cdot (1-\left[\sum_{t=i-T_{\text{np}}}^{i-1}m_{t}+\frac{m_{i}}{2}\right]\cdot \Delta )$$

A deeper analysis of this equation allows to solve the problem of hybrid DT model. Inside the Equation (3) we can separate the lost rate due to the NP-DT (*L_np_*) from the original true rate *n*, being:

(4) $$m_{i}=n_{i}-L_{i}^{\text{np}}\Rightarrow L_{i}^{\text{np}}=n_{i}\cdot \left[\sum_{t=i-T_{\text{np}}}^{i-1}m_{t}+\frac{m_{i}}{2}\right]\cdot \Delta$$

The quantity is characteristic of the DT type (*np*) and varies with the time position of the *i-th* interval. Equation (4) shows that the lost rate is the incoming true rate multiplied by the probability of a measured event in the previous τ*_np_* time interval.

### 3.2. Paralyzable Dead Time

Using the exponential distribution of time intervals between Poissonian random events occurring at a constant rate *n*, it is possible to show that the relation between measured rate and true rate in the case of P-DT is [1]:

(5) $$m=n\cdot \text{exp}(-n\cdot \tau_{p})$$

In the case of time dependent count rate the two values of the true rate *n* appearing in Equation (5) are different: the first one represents the true actual rate, the latter represents the true rate in the previous time intervals, source of DT losses. Taking into account the contribute of all previous *T_p_* intervals, and half weighting the *i-th* interval as in the case of NP-DT previously analyzed, we obtain the equation:

(6) $$m_{i}=n_{i}\cdot \text{exp}\left(-\left[\sum_{t=i-T_{p}}^{i-1}n_{t}+\frac{n_{i}}{2}\right]\cdot \Delta \right)$$

where *T_p_* is the number of intervals contained in τ*_p_*.

As before, we can separate the lost rate due to the P-DT (*L_p_*) from the original true rate *n*:

(7) $$m_{i}=n_{i}-L_{i}^{p}\Rightarrow L_{i}^{p}=n_{i}\cdot \left(1-\text{exp}\left(-\left[\sum_{t=i-T_{p}}^{i-1}n_{t}+\frac{n_{i}}{2}\right]\cdot \Delta \right)\right)$$

### 3.3. Hybrid Dead Time

In the hybrid DT model, introduced by Lee and Gardner [3], the DT configuration is a combination of P-DT and NP-DT. In constant regime the relation between true and observed count rates for this model is:

(8) $$m=\frac{n\cdot \text{exp}(-n\cdot \tau_{p})}{1+n\cdot \tau_{\text{np}}}$$

**Figure 1.**
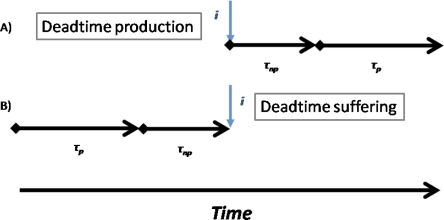
**(A)** Structure of the two DT produced in a hybrid DT model. **(B)** Time intervals rate that produces losses into the following *i-th* instant and type of DT losses actuated.

A detailed description and the derivation of this equation can be found in [3]. The count that arrives at the *i-th* interval generates a NP-DT followed by a P-DT, as shown in Figure 1(A). The two DT durations could be different.

In the time dependent count rate, we must consider which previous intervals produce losses into the *i-th* interval and the type of the actuated DT losses. Due to the sequence of produced DTs reported in Figure 1(B), the rates located inside the interval τ*_p_* produce P-DT losses, while the rates located inside the interval τ*_np_* produce NP-DT losses. Once identified the type losses produced by each previous interval we can write the equation for hybrid model valid for time dependent sources, starting from Equation (4) and Equation (7):

(9) $$m_{i}=n_{i}-{}^{*}L_{i}^{p}-L_{i}^{\text{np}}=n_{i}-n_{i}\cdot \left(1-\text{exp}\left(-\left[\sum_{t=i-T_{\text{np}}-T_{p}}^{i-T_{\text{np}}-1}n_{t}\right]\cdot \Delta \right)\right)-n_{i}\cdot \left[\sum_{t=i-T_{\text{np}}}^{i-1}m_{t}+\frac{m_{i}}{2}\right]\cdot \Delta$$

Note that the *i-th* interval generates NP-DT losses, so it must be considered only in the non paralyzable part of the equation. The symbol “*” indicates that last *i-th* term of Equation (7) is not considered.

### 3.4. Relation between Non Steady-State and Steady-State Equations

The non steady-state equation must be valid also in the steady-state, therefore imposing the steady-state condition in the Equations (3), (6) and (9) we must obtain the Equations (1), (5) and (8) respectively.

In fact in steady-state conditions *m_i_* and *n_i_* are constant series so their sum becomes the constant value multiplied by the number of repetitions and this multiplication factor multiplied with the time interval Δ gives the DT extension of the steady-state equation, τ*_np_* or τ*_p_*.

Moreover in the steady-state condition the size of the time interval (Δ) can be chosen as short as possible, so using the time precision of the readout system the losses produced inside the *i-th* interval should not be considered.

For the sake of clarity the mathematical steps that give Equation (8) starting from (9) are shown below:

(10) $$\begin{array}{l} m_{i}=n_{i}-n_{i}\cdot \left(1-\text{exp}\left(-\left[\sum_{t=i-T_{\text{np}}-T_{p}}^{i-T_{\text{np}}-1}n_{t}\right]\cdot \Delta \right)\right)-n_{i}\cdot \left[\sum_{t=i-T_{\text{np}}}^{i-1}m_{t}+\frac{m_{i}}{2}\right]\cdot \Delta \Rightarrow \\ m=n-n+n\cdot \text{exp}(-n\cdot \tau_{p})-n\cdot m\cdot \tau_{\text{np}}\Rightarrow \\ m=\frac{n\cdot \text{exp}(-n\cdot \tau_{p})}{\left(1+n\cdot \tau_{\text{np}}\right)} \end{array}$$

## 4. Montecarlo Simulations

A code, developed in Matlab, was performed to simulate the behavior of the three DT types. We found that, in this simulation approach, a sharp increase of the illumination from zero to a constant value, gives best information about the DT losses and the equation validity. The predictions of the three proposed equations (Equations (3), (6) and (9)) were compared with the results of the simulation.

We chose that the illumination value started with zero and remained zero for a time interval longer than DT. In this way Equations (3), (6) and (9) can be calculated in an iterative mode (*i* = *t*_0_, *i* = *t*_0_ + 1, *i* = *t*_0_ + 2, *i* = …), starting from the end of a zero illumination period, characterized by measured (*m_i_*) and true rates (*n_i_*) both equal to zero.

**Figure 2.**
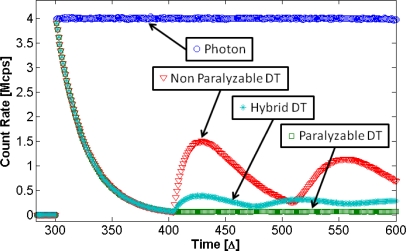
Histograms of rate *vs*. time; blue circle, simulated photon source rate; dark green square, P-DT model simulation; red triangle, NP-DT model simulation; cyan asterisk, hybrid DT model simulation.

Montecarlo simulation uses the Poisson statistics to generate the source count distribution, and the DT laws to determine which counts are measured and which counts are lost. In the zero illumination zone no count is introduced. In the constant illumination region a Poisson distribution was used to extract the random values of photons that should be put into the illumination time window. The time position of these photons was randomly chosen inside the illumination time window. The histogram of the simulated photon source count rate is shown in Figure 2. For the three DT simulations the three DT creation laws are used to determine the counted photons, the lost photons that arrive into DT, and the photons that produce or extend DT. Like in experimental setup, 1E6 acquisition windows were simulated. The histograms of this three DT simulations are reported in Figure 2. The time unit of the simulation is the interval duration Δ = 10 ns, the total DT extension was chosen of 105Δ for all three simulations.

The agreement between the simulations and the data calculated with the Equations (3), (6) and (9) is better than expected, being deviation less than 0.2%. The equation predictions are not plotted in Figure 2 because they overlap the simulation data.

In the simulation, the first 300Δ are defined without photons, after that the illumination source starts with a constant rate. In the following 105Δ, corresponding to the total DT duration, the shape of the three DT types are the same, because in this period, independently from the type of the occurring active DT, only one photon can be detected. Therefore this first part of the histogram shows the arriving time position of the first photons. This shape represents the probability to have no photon in illumination condition. This probability drastically decreases on increasing the time. After a time equal to the total DT the type of DT generates changes in the shape of the response. The P-DT that takes into account all lost counts shows a constant output rate, due to the constant lost incoming rate. The NP-DT shows an increase of counts due to the end of the DT of the first measured count not extended by the others photons. The hybrid model shows a behavior in the middle of the two fundamental DT types, according to the proportion of the DT component of hybrid model.

The P-DT reaches the steady-state value of Equation (5) after the first DT period, while the other DT types need few DT periods to reach their steady-state values, predicted from Equations (2) and (8). This is an important confirmation of the simulation goodness.

## 5. Experimental Validation

To test the features of the proposed new hybrid DT Equation (9) we used a Light Emitting Diode powered by a voltage which grows according to an exponential shape with a 0.05 μs time constant, and that falls down with a 100 μs time constant. The maximum photon rate that hit the detectors was about 500 kcps. The test device was a passive quenched SPAD with a measured 970 ns total hybrid DT [5]. In a constant illumination setup, at 500 kcps of photon rate, this device would be affected by 35% of losses. Instead the active quenching SPAD device, with a DT of 50 ns, at this rate can be considered free from losses, and it was used as reference counter.

Exposing the test device to a time dependent source we observed that the behavior changed with time. At the beginning the device was affected only by the negligible DT losses produced by the previous darkcount rate, 1 kcps, so the measured rate was very close to real one. Immediately after the measured rate fell down for the DT losses produced by the first detected rate. When the DT losses production reached the maximum value, the measured rate continued with a slope different from that produced by the reference counter.

The Equation (9) describes the relation between the true and the measured photon rates. Starting from the Equation (9) it is possible to extract the equations of real count rate and measured count rate, as a functions of previous true and measured count rates:

(11) $$n_{i}=\frac{m_{i}}{\text{exp}\left(-\left[\sum_{t=i-T_{\text{np}}-T_{p}}^{i-T_{\text{np}}-1}n_{t}\right]\cdot \Delta \right)-\left[\sum_{t=i-T_{\text{np}}}^{i-1}m_{t}+\frac{m_{i}}{2}\right]\cdot \Delta }$$

(12) $$m_{i}=\frac{n_{i}\cdot \left\{\text{exp}\left(-\left[\sum_{t=i-T_{\text{np}}-T_{p}}^{i-T_{\text{np}}-1}n_{t}\right]\cdot \Delta \right)-\left[\sum_{t=i-T_{\text{np}}}^{i-1}m_{t}\right]\cdot \Delta \right\}}{\left(1+n_{i}\cdot \frac{\Delta }{2}\right)}$$

**Figure 3.**
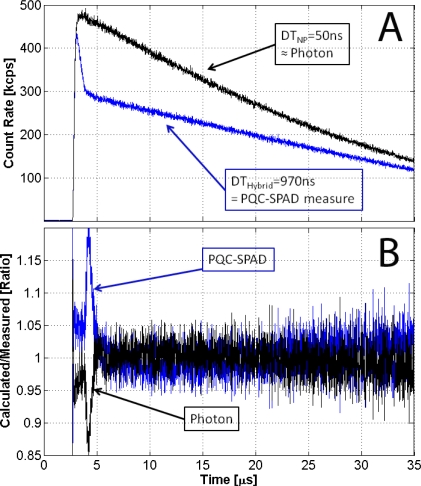
**(A)** black, true count rate, measured with active quenched SPAD; blue, measured count rate that suffer Hybrid DT losses (passive quenched SPAD). **(B)** Comparison between calculated rate and measured one; blue, calculated with Equation (12); black, calculated with Equation (11).

Assuming that during the dark state the count losses can be neglected, it is possible to evaluate the real true rate from the measured one using the Equation (11) and vice versa the measured rate from true rate using the Equation (12). In Figure 3 we compare the reference true rate with the predicted one using Equation (11), and the measured rate affected by hybrid DT losses with the predicted one using the Equation (12).

The difference between predicted and measured rate are in the first part of the light pulse because, due to the rapid change in the excitation rate, the device doesn’t show exactly the hybrid DT behavior but something close. After a time double the DT duration the predicted and the measured rates show a very good agreement, being deviation less than 5%.

## 6. Conclusions

The presented new approach for evaluating DT losses is able to show how the two elementary DT types and their combination works with time dependent sources. The theoretical derivation, the completely accordance with the Montecarlo simulation, the inclusion of classic DT equation, and the experimental validation are a well-round presentation of this three new equations, that could be useful in all counting system devices and application. Worth to note that our DT compensation technique requires only a simple data analysis process, apart from the type of time dependent source and without the necessity of developing complex electronic systems.

Our theoretical approach can be also useful to deduce new equations for other series or parallel combinations of P-DT and NP-DT. The presented equation can be also used to predict the behavior of many devices in time dependent applications, so allowing to select the most appropriate device.

In particular we plan to use this technique in the development of a new time resolved single-photon imaging sensor (TRIS) [8], which uses passive quenched SPAD devices and shared readout channels for many diodes. Using a passive quenched SPAD device, instead of active quenched one, we increase the fill factor of the device and using the presented correction technique we take advantage from the DT losses that characterize this device. In fact, especially in scared readout configuration, the reduced measured rate due to the DT losses allows to not saturate the readout electronics.
